# The Influence of Rehabilitation on Quality of Life in Breast Cancer Survivors: A Clinical Study

**DOI:** 10.3390/ijerph18168585

**Published:** 2021-08-14

**Authors:** Teresa Paolucci, Aristide Saggino, Francesco Agostini, Marco Paoloni, Andrea Bernetti, Massimiliano Mangone, Valter Santilli, Raoul Saggini, Marco Tommasi

**Affiliations:** 1 Department of Oral Medical Science and Biotechnology, G. D’Annunzio University of Chieti-Pescara, 66100 Chieti, Italy; teresa.paolucci@unich.it (T.P.); raoul.saggini@unich.it (R.S.); 2 Department of Medicine and Aging Sciences, G. D’Annunzio University of Chieti-Pescara, 66100 Chieti, Italy; aristide.saggino@unich.it (A.S.); marco.tommasi@unich.it (M.T.); 3 Department of Anatomical and Histological Sciences, Legal Medicine and Orthopedics, Sapienza University, 00185 Rome, Italy; marco.paoloni@uniroma1.it (M.P.); andrea.bernetti@uniroma1.it (A.B.); massimiliano.mangone@uniroma1.it (M.M.); valtersantilli@uniroma.it (V.S.)

**Keywords:** breast cancer, exercise, physiotherapy, MMPI-2, personality profile, pain

## Abstract

Background: Breast cancer survivors report negative impacts of cancer, augmented by specific vulnerabilities to body changes, negative self-assessment, and quality-of-life concerns. The main objective of our work was to test the effect of a rehabilitation program on breast cancer patients by evaluating the change in their physical well-being during an outpatient rehabilitation setting and, subsequently, in a home rehabilitation setting, considering the individual personality profile. Methods: Patients who underwent total mastectomy with breast prostheses or tissue expanders were enrolled. Outcome assessments (Pain, Quality of Life, Personality traits for the Minnesota Multiphasic Personality Inventory-2) before treatment (T0), at the end of the rehabilitative treatment (T1 = 10 sessions 2/week, one hour/each), and after two months of follow-up (T2) were performed. Results: The data of 38 included patients were analyzed. The quadratic trend of the Visual Analogue Scale can be explained by the fact that patients have a strong reduction in the perceived pain immediately after rehabilitation in the clinic. This reduction remains constant for the home period of the rehabilitation. The personality profiles of all the participants were substantially valid. Only three patients obtained scores higher than 65 points. Conclusions: The study evidenced that in the initial phase of the rehabilitation, psychological traits such as anxiety, depression, and preoccupation could have a strong association especially with the autonomous functions and the perceived physical symptoms. However, during the therapeutic process, this association decreased and these decrements were higher when patients performed their rehabilitation at home, in a more familiar and comfortable setting.

## 1. Introduction

Breast cancer (BC) is the most common cancer among women in developed countries, causing premature mortality, also presenting several disabling complications related to treatment [1]. BC survivors have reported negative impacts of cancer, augmented by vulnerabilities related to body changes, negative self-assessment, and quality of life (QoL) issues. Villa et al. showed how physical function size, body image, financial worries, and symptoms worsened after surgery, while emotional function (anxiety) and prospects improved [2]. It could be hypothesized that subsequent rehabilitation and psychological support interventions are very important phases following oncological surgeries for different sequelae (pain, function of the operated limb, posture, body-image, and QoL) [3,4,5]. Chemotherapy/radiotherapy treatments also have a significant impact on the QoL and experience cognitive decline [6,7,8]. It is very important that the BC patient does not abandon or renounce rehabilitation treatments even during chemotherapy/radiotherapy [9,10]. On the other hand, cancer rehabilitation is often underutilized in BC survivors, at the expense of its importance and potential [11]. Mood disorders accompany BC survivors throughout most of their care pathways. Depression is considered a psychological factor negatively associated with QoL, and anxiety increases vulnerability to cognitive impairment after chemotherapy [12]. QoL after BC has worsened, highlighting the need for interdisciplinary work dedicated to recovering mental health and function with an increased focus on rehabilitation [13]. Surgery is the first line against BC and depends on many cancer factors and what is acceptable to patients. Nowadays, breast-conserving surgery is preferred over total mastectomy because it leads to better body image results, outlook, and fewer side effects [14]. Surgery can be accompanied by serious complications such as shoulder dysfunction, upper limb pain, post-mastectomy syndrome, chemotherapy-induced peripheral neuropathy, axillary cord, lymphedema, and postural imbalance [9]. Breast reconstruction, during/after surgery, can avoid psychological discomfort to the patient and reduce pain [15]. Usually, surgery or adjuvant therapies are accompanied by suffering and negative emotions, which affect the QoL, especially in cancer patients. Emotional disturbances alter the perception of pain and the painful part of the body is often avoided, both from a tactile and visual point of view, and excluded from motor organization [16,17]. Because of the sequelae, which BC survivors often report, and the delicate interplay between pain and psyche, a key aspect of the rehabilitation is an individualized approach that meets patients’ needs while taking emotional aspects into account [18,19]. The choice of the rehabilitation setting is also important: a single rehabilitation setting would seem to favor a better therapeutic alliance between BC survivors and the physiotherapist [7]. Considering these premises, we hypothesized that BC survivors may demonstrate a different response in terms of pain and QoL during the rehabilitation process performed in the rehabilitation clinic, in a first phase, and at home, in a second phase. The main objective of our work was to test the effect of a rehabilitation program on BC patients by evaluating the change in their physical well-being (QoL) during an outpatient rehabilitation setting and, subsequently, in a home rehabilitation setting, considering the individual personality profile.

## 2. Materials and Methods

A clinical study (July–September 2019) took place at the rehabilitation out-patient clinic of University Hospital Umberto I, Sapienza University of Rome (Italy). The study protocol was approved by the Ethics Committee of Sapienza University of Rome (N° 4985).

Patients were enrolled in the study after an evaluation by a rehabilitation physician considering the following inclusion criteria: total mastectomy with breast prostheses or tissue expanders; age 18–60 years; body mass index (BMI) < 30; no cognitive dysfunctions (MMSE > 24); no lymphedema. The exclusion criteria were: lymphangitis, metastasis, surgical complications of the intervention; neurological deficits and complications; important shoulder problems before the BC-surgery; web axillary syndrome; other or previous physiotherapy; psychiatric disorders.

All patients signed an informed consent, after receiving detailed information about the study’s aim and procedures for the Declaration of Helsinki. The rights of human subjects involved in the study were protected. This study protocol was developed in accordance with the Trend Guidelines [20].

### 2.1. Measures

Outcome assessments were performed before treatment (T0 = baseline), at the end of the rehabilitative treatment (T1 = 10 sessions 2/week, one hour/each), and after two months of follow-up (T2). Because the first part of the rehabilitative therapy was conducted in the clinic, and the second part continued at home, we defined two time periods for the rehabilitation: (a) the clinical period (T0–T1) and the (b) home period (T1–T2).

Age, height, weight, and BMI were collected by the rehabilitation physician at the first evaluation. A clinical evaluation of the shoulder Range of Motion (ROM) on the operated side was performed. Patients were referred, by the oncologist, or by the surgeon or general practitioner, after the surgery (time from intervention 9.67 ± 5.12) to the psychiatric examination, which did not have the possibility to carry out a pre-surgical evaluation.

### 2.2. Evaluation Scales

#### 2.2.1. Pain

The Visual Analogue Scale (VAS) was administered as a simple, robust, sensitive, and reproducible instrument, which enables the patients to express their pain intensity as numerical values from 0 to 10 cm [21,22].

#### 2.2.2. QoL

The European Organization for Research and Treatment of Cancer Quality of Life core questionnaire (EORTCQLQ-C30) [23,24,25] is the global cancer-specific questionnaire that is used to examine the health-related QoL among patients with cancer. This is a 30-item core questionnaire, used to assess the physical and psychosocial functioning and symptom experiences. The QLQ consists of a scale for Global Health Status-revised (QL2) and a Global Symptom Index (GSI). The GIF and GSI were used, together with the QL2, to analyze the variations in the QoL, the level of functioning, and the severity of physical symptoms during the rehabilitation. The acquired scores of each scale are spread in the 0–100 domain.

#### 2.2.3. Personality Traits

The MMPI-2 is one of the most widely used personality questionnaires used for assessing personality traits or psychological syndromes. Each item of the MMPI-2 describes a typical sensation, thought, or behavior associated with a specific personality trait or syndrome, and patients responded to each item using “true” or “false” if they had or not, respectively, that specific characteristic. The questionnaire is composed of 567 items and it comprises 10 basic clinical scales (Hypochondriasis—HS, Depression—D, Hysteria—HY, Psychopathic Deviate—PD, Masculinity-Femininity—MF, Paranoid—PA, Psychasthenia—PT, Schizophrenia—SC, Hypomania—MA, and Social Introversion—SI scale) and three basic validity scales (Lie—L, Frequency—F, and Correction—K scale). Validity scales estimated the tendency of participants to improve their characteristics for social desirability (Lie), to exaggerate their symptoms or traits (Frequency), or to hide some specific problems (Correction). In addition, we included in the analysis some content scales that are the Anxiety (ANX), Fears (FRS), Obsessiveness (OBS), Depression (DEP), Health Concerns (HEA), and Negative Treatment Indicators (TRT) scales to have further confirmation of the probable connection between the personality traits and the measure of pain, level of functioning, and severity of physical symptoms. The TRT scale is normally used to test the existence of a possible resistance against a psychotherapeutic treatment. In this case, we used this scale to test the possible resistance against the rehabilitative procedure. We used the Italian adaption of the MMPI-2 [26,27]. MMPI-2 was administered only at T0, considering the personality already structured in the adult person and, in any case, not modifiable in a few months.

#### 2.2.4. Rehabilitative Treatment

The patients underwent ten single rehabilitation treatment sessions (60 min/session; 2/week). Then, during the rehabilitation process, the patients were taught active recovery exercises for the articulation of the shoulder on the operated side, which they could repeat independently at home at the end of the cycle of ten sessions with the physiotherapist. For greater protection, patients were given an illustrative pamphlet with the exercises to continue to be performed at home at least twice a week. The physiotherapists tailored the rehabilitation to the patients’ functional problems. A previously validated rehabilitation protocol was considered [28,29].

#### 2.2.5. First Phase

Exercises carried out with the physiotherapist in the first phase of outpatient rehabilitation: in supine position for relaxation and diaphragmatic breathing; exercises in the supine position of postural re-alignment with the use of a small cylinder on the line of the vertebral spines; exercises of decoaptation and co-activation of the glenohumeral joint; exercises to stretch the pectoral muscles, subscapularis muscle; exercises to mobilize the scapulo-thoracic joint; cervical pumping; active exercises in the mirror in a sitting position, for the recovery of the range of motion of the shoulder (for example, favoring flexion with the use of the stick); isometric strengthening exercises in flexion, abduction, and adduction of the arms also with the use of rubber bands; Codman’s pendulum; balance exercises standing in front of the mirror. All proposed exercises were repeated, starting from 10 repetitions for 3 times (adapting the increase in performance to the patient’s compliance and resistance, progressing gradually during the sessions). Before starting the session, the patients performed a short 10–15 min warm-up.

#### 2.2.6. Second Phase

At the end of the rehabilitation treatment, patients were given an illustrative pamphlet with the exercises to be performed at home and with the indication to walk as much as possible at least 30 min almost every day. Exercises during the first month of observation/home-based exercise were given twice a week and continued in the second month with once a week. The exercises indicated aimed at the active mobilization of the shoulder such as flexion and extension with the use of the stick, or favor abduction by going up the wall with the hand in a lateral position; exercises of rotation of the arms in 90° abduction; exercises in adduction; twisting exercises for the trunk in sitting and standing position; mirror active cervical mobilization exercises. The patients had a rehabilitation diary available to record adherence to treatment.

## 3. Results

### 3.1. Statistical Analyses

Sample size was not predefined but determined by the period of observation of the study, that is, 1 year.

A self-report questionnaire was used to analyze the evolution of pain and QoL, and the severity of physical symptoms in patients. In addition, we measured personality trait to analyze their mediation effect on the evolution of the pain, functioning, and physical symptoms during the rehabilitation.

#### 3.1.1. Frequencies and Descriptive

Frequencies and descriptive statistics were provided for demographic and biometric data. Descriptive data were mean, standard deviation, minimum and maximum values, skewness, and kurtosis. Skewness and kurtosis values between −2 and 2 indicate a good distribution of the data [30].

#### 3.1.2. Time Series Analysis

We analyzed the trend of the rehabilitation effects on the measures of pain, functioning, and severity of physical symptoms (VAS, QL2, GIF, and GSI) using the regression analysis with orthogonal polynomial coefficients. The polynomial coefficients for the linear trend were −1, 0, and 1, and the coefficients for the quadratic trend were −1, 2, and −1. The *t*-test was applied to determine whether the linear or quadratic trend was significant. Positive (negative) t-values for the linear trend indicated a constant increasing (decreasing) effect from T0 to T2, and positive (negative) t values for the quadratic trend indicated a maximum (minimum) effect in the middle (T1) of the time series [31]. We also estimated the effect size in relation to significant t-values. The effect size is low at <0.5, medium at <0.8, and high at >0.8 [32,33].

#### 3.1.3. Moderation Effects in Time Series of Psychological Traits

**Figure 1:**
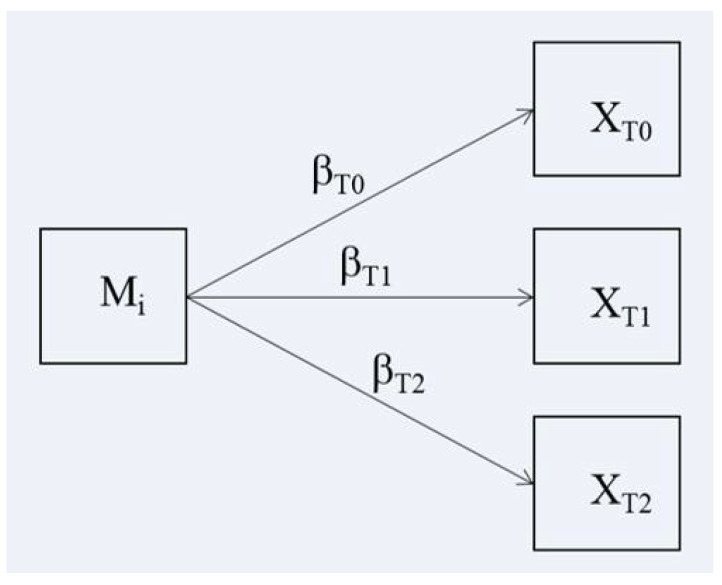
Path diagram of the regression models for testing moderation effect in time series. Mj: mediation variable of the j-th subject; X_Ti_: variable measured in the i-th period of time (T0, T1, and T2); β_Ti_: coefficient of the slope associated to X_Ti_.

The moderation effects were tested using regression models with the following equation:

X_Ti_ = β_0_ + βε_Ti_ M_j_ + ε_j_ (1)

where Mj is the moderating variable (in this case, the psychological trait) of the j-th subject, X_Ti_ is the variable (VAS, QL2, GIF, and GSI) measured in the i-th period of time (T0, T1, and T2) or the criterion, βTi is the coefficient of the slope associated with X_Ti_, β_0_ is the intercept, and ej the random error. The variation in βTi indicates the strength of the relationship between M and X_Ti_. With the mediation analysis, it is possible to analyze the variation in this strength from T0 to T2. In particular, if the strength increases, then we should have βT0 < βT1 < βT2. If the strength decreases, then we should have βT0 > βT1 > βT2. If the slopes vary for each time period, then there is an interaction between M and X_Ti_ or, in other words, there is a moderation of M_j_ on X_Ti_. Figure 1 shows the regression models to test moderation in time series. To test the moderation hypothesis, it is necessary to test the significance of the differences between βT0, βT1, and βT2. We analyzed the mediation effect in relation to the clinical period (βT1–βT0) and to the home period (βT2–βT1). Positive β differences indicate an increment in the relation between the criterion and the psychological trait; negative β differences indicate a decrement in the relation between the criterion and the psychological trait. All statistical analyses were performed in R-studio, version 1.2.1335 and M-plus, 7th edition [34].

### 3.2. Patients’ Data

During a period of 12 months, 58 patients were observed. In addition, 41 patients were enrolled into the study and assigned to the intervention. During treatment, 3 patients dropped out for personal problems. None complained about pain. The data of 38 included patients were analyzed (Table 1).

### 3.3. Effects of Rehabilitation on Pain, Functioning, and Severity of Physical Symptoms

**Figure 2:**
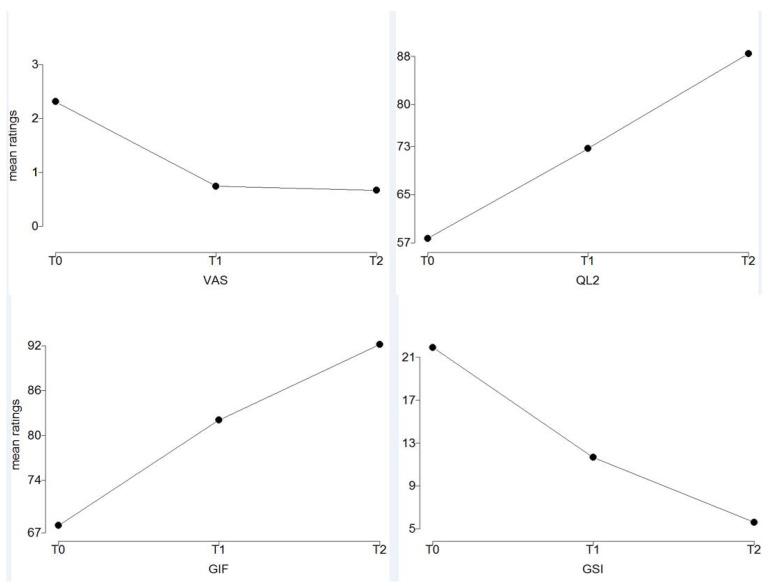
Time series of perceived pain (VAS), quality of life (QL2), global index of functioning (GIF), and global symptom index (GSI) of patients. T0 is the clinical pre-period; T1 the clinical post-period; T2 the home post-period.

The linear and quadratic components of the VAS were significant (*p* < 0.001), while only the linear components of QL2, GIF, and GSI were significant (*p* < 0.001) (Table 2, Figure 2).

For the QL2, GIF, and GSI, there was a prolonged positive effect of the rehabilitation, both for the clinic and the home period. Table 3 shows the descriptive statistics for the MMPI-2 scales. The scale is divided into three groups: validity, clinical/personality, and content scales group. The personality profiles of all the participants are substantially valid. Only three patients obtained scores higher than 65 on the L scale and only one on the F scale. All skewness values and nearly all kurtosis values (except one for the MF scale) were between −2 and 2, as a good distribution of the data.

### 3.4. Rehabilitation and Psychological Traits

Table 4 shows moderation effects of the psychological traits on VAS, QL2, GIF, and GSI. The β-coefficients for each period of time (T0, T1, and T2) are reported with their standard error and with the corresponding standardized β-values (β′). The moderating effects were tested by calculating the differences between the β coefficients for the clinic (β_T1_–β_T0_) and home period (β_T2_–β_T1_). The t-values and relative probabilities p(t) for each β differences are reported in Table 4. In particular, with respect to the primary outcome, the results suggest a moderation effect of the HS and HY scale for the GIF. There is also a constant association with the QoL2 for ANX, OBS, DEP, and TRT of content scales of the MMP.

## 4. Discussion

The aim of our research was to test the influence of a rehabilitation program on BC survivors with respect to their QoL considering the individual personality profile. Usually, it is not possible to perform prolonged treatments in the rehabilitation outpatient setting; therefore, an early discharge is required with an acceptable achievement of the rehabilitation goal, and it is not possible to renew the rehabilitation cycle, hence the importance of giving the patient instructions to be able to continue the treatment in the home environment independently and safely, with “remote” support from the physiotherapist. Our results appeared to be encouraging; the rehabilitation plan reduced the perceived pain and increased the perceived quality and the level of functioning, and these effects were persistent also when patients must perform their rehabilitation at home. The quadratic trend of the VAS can be explained by the fact that patients have a strong reduction in the perceived pain immediately after the rehabilitation in clinic; this reduction remained constant for the home period of the rehabilitation.

The literature indicates that rehabilitation exercise is effective in improving the QoL in patients who have survived cancer [35,36]; in addition, referring to a supervised rehabilitation program for the fatigue reduction [37], there are no studies that have specifically addressed the rehabilitation setting impact and the psycho-emotional implications linked to the patient’s personality. Kirkham et al. reported how adherence to supervised exercise, delivered in a clinical setting, varies between BC patients and treatments. Additionally, behavioral strategies and individualization in exercise prescriptions to improve adherence are particularly important for subsequent courses of chemotherapy after treatment [38]. Furthermore, pain and QoL are closely linked in BC with a negative trend [39] and creates a psycho-emotional distress, which often aggravates the patients’ state of anxiety and depression. The absence of a significant relation between the standardized coefficient of MMPI and VAS scale, as indicated by our results, may be explained by the fact that the VAS scale measures the level of perceived pain, which is a physical sensation [26,27]. Therefore, the psychological characteristics of individuals do not have a significant predictive validity on its intensity. However, as our results suggest, for the QLQ measures, it is possible to see some associations between psychological characteristics and the QL2, GFI, and GSI scales. These associations, indicated by the significance of regression coefficients, evidence the different patterns of association between the psychological traits and the level of QoL. First, the clinical scales showed no significant predictive efficacy, except the HS and HY scale for the GIF. The HS scale measures the excessive preoccupation of patients about their body and physical diseases or a general preoccupation about their health, in the absence of any real cause or pathology [23]. The HY scale measures people’s tendency to develop sensory or motor diseases without any organic origin and the tendency to negate the presence of problems in their life. Then, these two scales had a negative slope with the GIF, indicating that patients with high levels of hypochondria and hysteria tend to show a low level of functioning, as shown in Table 4. However, the magnitudes of these slopes decreased during the period of rehabilitation, reaching a minimum level at T2. Second, we could consider that the preoccupation for the health and the presence of sensory or motor diseases decreased during the rehabilitation. Lastly, there were some content scales of the MMPI that showed a constant association with the QoL: the MMPI scales were ANX, OBS, DEP, and TRT [24]. The ANX scale measured the level of general anxiety, including the classical physical symptoms (tachycardia and lack of breathing); the OBS scale measured the difficulty to take decisions, tendency to develop compulsive behaviors, and excessive preoccupations; the DEP scale measures the tendency to develop depressive thoughts, negative emotions, and sense of hopelessness; the TRT scale measures people’s oppositional attitude toward physicians or medical treatments and the tendency to believe that nobody can help them [23,24]. The results showed that, during the rehabilitation, there was a significant decrement in the association between these MMPI scales and the QL2, and the GIF, GSI, and moderation analysis showed that the difference between coefficients was more significant during the home period rehabilitative treatment. Therefore, during the period in which patients have to carry out their rehabilitative therapy at home, the improvement of their physical condition reduced the impact that negative psychological traits, such as anxiety, depression, and preoccupation, had on their perceived QoL level and increased their compliance toward the rehabilitative therapy. The efficacy of home-based multidimensional programs for BCs have gained an ever-greater emphasis in survivorship care to maximize QoL for their successful transition to rehabilitation and normal life. İzci et al. underlined that the BC patients with extraversion personality traits have lower levels of anxiety and depression, maintaining their QoL, whereas the patients with higher neuroticism scores may have more QoL [40]. Therefore, the rehabilitation not only improves patient’s health and functioning, but it can also have a positive effect on their psychological conditions, which, in turn, could have a supportive role in the rehabilitative process. The principal strength of our study is that it tests the influence of the rehabilitative program in two different settings, outpatient and home, and that it is the first study to evidence an association between psychological traits and the effects of the physiotherapeutic process on BC patients’ well-being. Our study presents some limitations. The first limitation was the small sample size, even if size dimensions were determined by the availability of patients to participate in the rehabilitative plan. Furthermore, the study lacked an effective observation and follow-up period without any rehabilitation suggestions. Not being a randomized controlled trial, we did not pre-calculate the sample size. Other limitations were that psychological characteristics were not measured in different periods of time. However, the MMPI is a questionnaire with many scales and many items, and it is not feasible for a repeated administration. The use of short-form questionnaires could probably represent a more practical solution for time series studies.

## 5. Conclusions

In conclusion, our study evidenced that the rehabilitation in BCs has a positive effect, because patients showed a reduction in pain and an increment in the QoL, with an increment in autonomous functioning and a decrement in physical symptoms. The study evidenced that in the initial phase of the rehabilitation, psychological traits such as anxiety, depression, preoccupation, and, in some cases, hypochondria and hysteria could have a strong association, especially with the autonomous functions and the perceived physical symptoms. Further studies are needed, with a larger sample and a longer follow-up, in order to better highlight the effects of rehabilitation intervention on the quality of life in breast cancer survivors.

## Figures and Tables

**Table 1:** Frequencies and descriptive statistics (mean, standard deviation, minimum value, maximum value, skewness, and kurtosis) for demographic and biometric data.

Descriptive Variables	Mean ± Sd	Min, Max	Skewness	Kurtosis
Age	52.00 ± 6.50	36, 60	−0.53	−0.51
BMI	23.95 ± 3.61	18, 32	0.64	−0.56
TfI (months)	9.67 ± 5.12	2, 24	0.94	0.89
**Frequencies**	**Type**	**N**	**%**	
Family status	Single	8	21.05	
	Conjugated	28	73.68	
	Married	1	2.63	
	Divorced	1	2.63	
Profession	Occupied	29	74.36	
	Homeworking	4	10.53	
	Unemployed	5	13.16	
Education	Secondary school	5	13.16	
	High school	25	65.79	
	University	8	21.05	

Sd: standard deviation; Min: minimum; Max: maximum; BMI: body mass index; TfI: time from intervention.

**Table 2:** Regression analysis of linear and quadratic trends for time series (T0, T1, and T2) of VAS, QL2, GIF, and GSI scores.

Scales for Rehabilitation Assessment	Trend	Estimated Coefficient	Std. Error	t Value	Pr(>|t|)	Cohen’s d	Effect Size Level
VAS	lin.	−1.641	0.222	−7.395	**<0.001**	2.368	high
	quadr.	−1.487	0.187	−7.959	**<0.001**	2.549	high
QL2	lin.	30.718	3.757	8.176	**<0.001**	2.618	high
	quadr.	−0.821	4.193	−0.196	0.846		
GIF	lin.	24.230	3.530	6.865	**<0.001**	2.199	high
	quadr.	3.974	3.405	1.167	0.250		
GSI	lin.	−16.282	2.634	−6.182	**<0.001**	1.980	high
	quadr.	−4.179	2.434	−1.717	0.094		

Significant *p* values are in bold. VAS: Visual Analog Scale of pain; QL2: Quality-of-Life scale (revised); GIF: Global Index of Functioning; GSI: Global Symptom Index. Lin.: linear trend; quadr.: quadratic trend.

**Table 3:** Descriptive statistics (mean, standard deviation, minimum value, maximum value, skewness, and kurtosis) for MMPI-2 scales.

MMPI SCALES		Mean	Sd	Skewness	Kurtosis
**Validity Scales**	**Lie (L)**	55.667	7.593	0.093	−0.541
	**Frequency (F)**	51.077	7.600	0.315	−1.042
	**Correction (K)**	48.385	8.780	−0.148	−0.777
**Clinical/Personality Scales**	**Hypochondriasis (HS)**	62.359	12.162	0.486	−0.487
	**Depression (D)**	56.769	10.963	0.726	0.252
	**Hysteria (HY)**	57.462	12.185	0.625	−0.032
	**Psychopathic Deviate (PD)**	52.949	8.516	−0.414	1.166
	**Masculinity-Femininity (MF)**	51.615	11.502	1.372	4.100
	**Paranoid (PA)**	51.667	8.106	0.203	−0.224
	**Psychasthenia (PT)**	50.795	8.865	0.345	−0.386
	**Schizophrenia (SC)**	54.333	6.698	0.433	−0.844
	**Hypomania (MA)**	45.385	9.101	0.705	−0.039
	**Social Introversion (SI)**	50.103	10.694	0.348	−0.529
**Content Scales**	**Anxiety (ANX)**	55.513	9.660	0.017	−0.665
	**Fears (FRS)**	55.205	10.360	0.015	−0.791
	**Obsessiveness (OBS)**	48.256	7.351	0.124	−0.937
	**Depression (DEP)**	51.897	8.804	0.274	−0.633
	**Health Concerns (HEA)**	59.949	11.693	0.092	−1.067
	**Negative Treatment Indicators (TRT)**	51.513	11.541	0.077	−0.827

Sd: standard deviation.

**Table 4:** Unstandardized and standardized **β** coefficients for each period of time (T0, T1, and T2), and **β** differences for clinic and home period with corresponding t values and probabilities.

		Time Periods						Mediation Analysis					
		T0		T1		T2		Clinic Period			Home Period		
	MMPI Scales	β _T0_ (s.e.)	β′_T0_	β _T1_ (s.e.)	β′_T1_	β _T2_ (s.e.)	β′_T2_	β_T1_–β_T0_ (s.e.)	t	p(t)	β_T2_–β_T1_ (s.e.)	t	p(t)
**QL2**	HS	−0.496 (0.307)	−0.251	−0.559 (0.301)	−0.285	−0.225 (0.175)	−0.202	−0.063 (0.186)	−0.340	0.734	0.335 (0.264)	1.269	0.204
	D	−0.445 (0.344)	−0.203	−0.633 (0.334)	**−0.290**	−0.023 (0.198)	−0.019	−0.187 (0.205)	−0.913	0.361	0.609 (0.282)	2.161	**0.031**
	HY	−0.499 (0.306)	−0.253	−0.548 (0.301)	−0.279	−0.132 (0.177)	−0.119	−0.048 (0.186)	−0.259	0.796	0.416 (0.260)	1.599	0.110
	PD	0.150 (0.452)	0.053	0.323 (0.446)	0.115	−0.234 (0.252)	−0.147	0.173 (0.265)	0.653	0.514	−0.558 (0.374)	−1.493	0.136
	MF	−0.303 (0.331)	−0.145	−0.294 (0.329)	−0.142	−0.038 (0.189)	−0.032	0.008 (0.197)	0.042	0.967	0.256 (0.281)	0.912	0.362
	PA	−0.094 (0.475)	−0.032	−0.675 (0.459)	−0.229	−0.275 (0.264)	−0.164	−0.581 (0.264)	−2.198	**0.028**	0.400 (0.398)	1.004	0.315
	PT	−0.129 (0.434)	−0.048	−0.444 (0.426)	−0.165	0.114 (0.244)	0.075	−0.314 (0.251)	−1.252	0.211	0.558 (0.358)	1.559	0.119
	SC	−0.386 (0.572)	−0.107	−0.676 (0.561)	−0.189	0.024 (0.324)	0.012	−0.290 (0.336)	−0.864	0.388	0.700 (0.475)	1.472	0.141
	MA	**0.824 (0.402)**	**0.312**	0.447 (0.414)	0.17	−0.038 (0.239)	−0.026	−0.377 (0.242)	−1.557	0.119	−0.485 (0.351)	−1.383	0.167
	SI	−0.281 (0.357)	−0.125	−0.451 (0.350)	−0.202	0.106 (0.202)	0.084	−0.170 (0.211)	−0.809	0.419	0.557 (0.293)	1.904	0.057
	ANX	**−0.969 (0.367)**	**−0.389**	**−1.067 (0.357)**	**−0.431**	−0.193 (0.223)	−0.137	−0.098 (0.234)	−0.419	0.676	0.874 (0.308)	2.836	**0.005**
	FRS	−0.626 (0.358)	−0.269	−0.604 (0.356)	−0.262	−0.274 (0.205)	−0.209	0.021 (0.219)	0.098	0.922	0.331 (0.311)	1.063	0.288
	OBS	**−1.014 (0.498)**	**−0.310**	**−1.411 (0.469)**	**−0.434**	−0.192 (0.294)	−0.104	−0.398 (0.302)	−1.316	0.188	1.219 (0.400)	3.049	**0.002**
	DEP	−0.623 (0.426)	−0.228	**−1.083 (0.398)**	**−0.399**	−0.246 (0.244)	−0.160	−0.460 (0.247)	−1.862	0.063	0.837 (0.346)	2.417	**0.016**
	HEA	−0.349 (0.325)	−0.170	−0.540 (0.315)	−0.264	**−0.349 (0.177)**	**−0.300**	−0.190 (0.192)	−0.993	0.321	0.191 (0.278)	0.688	0.492
	TRT	−0.603 (0.320)	**−0.289**	**−1.072 (0.283)**	**−0.518**	**−0.384 (0.178)**	**−0.327**	−0.469 (0.182)	−2.581	**0.010**	0.688 (0.261)	2.635	**0.008**
**GIF**	HS	**−0.574 (0.273)**	**−0.319**	**−0.390 (0.196)**	**−0.304**	−0.114 (0.109)	−0.165	0.184 (0.211)	0.872	0.383	0.276 (0.185)	1.494	0.135
	D	−0.550 (0.307)	−0.276	**−0.554 (0.210)**	**−0.389**	−0.089 (0.122)	−0.116	−0.004 (0.236)	−0.015	0.988	0.465 (0.197)	2.358	**0.018**
	HY	**−0.628 (0.269)**	**−0.350**	**−0.531 (0.187)**	**−0.414**	**−0.234 (0.104)**	**−0.340**	0.097 (0.212)	0.459	0.646	0.297 (0.184)	1.619	0.105
	PD	0.470 (0.405)	0.183	0.200 (0.292)	0.109	−0.128 (0.156)	−0.130	−0.271 (0.301)	−0.900	0.368	−0.327 (0.266)	−1.229	0.219
	MF	−0.235 (0.302)	−0.124	−0.114 (0.217)	−0.084	0.051 (0.116)	0.07	0.122 (0.224)	0.543	0.587	0.164 (0.199)	0.825	0.409
	PA	−0.038 (0.432)	−0.014	−0.486 (0.299)	−0.252	−0.197 (0.162)	−0.191	−0.448 (0.311)	−1.440	0.150	0.289 (0.281)	1.028	0.304
	PT	−0.406 (0.390)	−0.164	−0.392 (0.275)	−0.222	−0.061 (0.151)	−0.065	0.014 (0.292)	0.049	0.961	0.331 (0.255)	1.295	0.195
	SC	−0.582 (0.515)	−0.178	**−0.766 (0.353)**	**−0.328**	−0.099 (0.200)	−0.079	−0.184 (0.385)	−0.477	0.633	0.667 (0.328)	2.033	**0.042**
	MA	0.583 (0.374)	0.243	0.181 (0.273)	0.105	0.067 (0.147)	0.073	−0.402 (0.277)	−1.454	0.146	−0.114 (0.253)	−0.449	0.653
	SI	−0.412 (0.321)	−0.202	−0.351 (0.227)	−0.240	0.100 (0.124)	0.128	0.061 (0.242)	0.254	0.799	0.451 (0.204)	2.216	**0.027**
	ANX	**−0.920 (0.332)**	**−0.406**	**−0.644 (0.238)**	**−0.398**	−0.238 (0.134)	−0.275	0.276 (0.264)	1.044	0.296	0.406 (0.230)	1.764	0.078
	FRS	−0.591 (0.325)	−0.280	−0.258 (0.238)	−0.171	−0.091 (0.129)	−0.112	0.333 (0.244)	1.363	0.173	0.167 (0.221)	0.757	0.449
	OBS	**−1.079 (0.444)**	**−0.362**	**−0.869 (0.311)**	**−0.409**	−0.175 (0.180)	−0.154	0.210 (0.350)	0.601	0.548	0.693 (0.294)	2.358	**0.018**
	DEP	**−0.767 (0.379)**	**−0.309**	**−0.864 (0.248)**	**−0.487**	**−0.289 (0.145)**	**−0.303**	−0.096 (0.293)	−0.328	0.743	0.575 (0.246)	2.34	**0.019**
	HEA	−0.495 (0.289)	−0.265	−0.374 (0.205)	−0.280	−0.170 (0.111)	−0.237	0.121 (0.220)	0.551	0.581	0.204 (0.195)	1.047	0.295
	TRT	**−0.752 (0.279)**	**−0.397**	**−0.767 (0.179)**	**−0.567**	−0.165 (0.113)	−0.227	−0.015 (0.224)	−0.066	0.947	0.602 (0.175)	3.435	**0.001**
**GSI**	HS	**0.445 (0.226)**	**0.300**	0.183 (0.166)	0.174	0.010 (0.101)	0.016	−0.262 (0.170)	−1.539	0.124	−0.173 (0.109)	−1.583	0.113
	D	0.337 (0.258)	0.205	0.173 (0.185)	0.148	0.024 (0.112)	0.035	−0.164 (0.193)	−0.850	0.395	−0.149 (0.123)	−1.211	0.226
	HY	0.396 (0.228)	0.268	0.188 (0.165)	0.179	0.131 (0.099)	0.208	−0.208 (0.172)	−1.211	0.226	−0.057 (0.110)	−0.508	0.611
	PD	−0.572 (0.327)	−0.270	−0.125 (0.240)	−0.083	0.031 (0.144)	0.034	0.447 (0.240)	1.862	0.063	0.156 (0.159)	0.980	0.327
	MF	**0.476 (0.239)**	**0.303**	0.245 (0.174)	0.220	0.115 (0.105)	0.172	−0.230 (0.182)	−1.267	0.205	−0.130 (0.117)	−1.110	0.267
	PA	−0.072 (0.356)	−0.032	0.398 (0.245)	0.252	0.250 (0.146)	0.264	0.470 (0.252)	1.864	0.062	−0.148 (0.167)	−0.883	0.377
	PT	0.172 (0.325)	0.085	0.042 (0.231)	0.029	−0.023 (0.139)	−0.026	−0.130 (0.240)	−0.541	0.588	−0.065 (0.154)	−0.422	0.673
	SC	0.352 (0.427)	0.131	0.210 (0.304)	0.110	0.032 (0.184)	0.028	−0.141 (0.318)	−0.445	0.656	−0.179 (0.202)	−0.882	0.378
	MA	−0.468 (0.308)	−0.236	−0.165 (0.224)	−0.117	−0.033 (0.135)	−0.039	0.304 (0.229)	1.323	0.186	0.132 (0.149)	0.887	0.375
	SI	0.101 (0.270)	0.06	0.016 (0.192)	0.013	−0.183 (0.111)	−0.255	−0.085 (0.199)	−0.428	0.669	−0.199 (0.124)	−1.602	0.109
	ANX	**0.686 (0.278)**	**0.368**	0.351 (0.205)	0.265	0.141 (0.125)	0.177	−0.335 (0.214)	−1.564	0.118	−0.210 (0.138)	−1.525	0.127
	FRS	0.491 (0.267)	0.282	0.071 (0.197)	0.058	−0.033 (0.119)	−0.045	−0.420 (0.195)	−2.156	**0.031**	−0.104 (0.131)	−0.796	0.426
	OBS	**0.885 (0.366)**	**0.361**	**0.534 (0.265)**	**0.307**	0.096 (0.167)	0.092	−0.350 (0.285)	−1.230	0.219	−0.438 (0.173)	−2.538	**0.011**
	DEP	**0.639 (0.312)**	**0.312**	**0.448 (0.221)**	**0.308**	0.175 (0.137)	0.201	−0.190 (0.240)	−0.792	0.428	−0.273 (0.149)	−1.831	0.067
	HEA	0.410 (0.238)	0.266	0.202 (0.172)	0.184	0.063 (0.105)	0.096	−0.209 (0.179)	−1.163	0.245	−0.139 (0.115)	−1.205	0.228
	TRT	**0.730 (0.221)**	**0.467**	**0.542 (0.155)**	**0.489**	0.171 (0.103)	0.257	−0.187 (0.182)	−1.027	0.305	−0.371 (0.103)	−3.617	**0.000**

β′ is standardized coefficient; p(t) = probability associated with the t values; s.e. = standard errors. Significant β_T0_, β_T0_, and p(t) are in bold types.

## Data Availability

All data are shown in the manuscript.
